# Parental exposure to heat waves improves offspring reproductive investment in *Tetranychus urticae* (Acari: Tetranychidae), but not in its predator, *Phytoseiulus persimilis* (Acari: Phytoseiidae)

**DOI:** 10.1002/ece3.10748

**Published:** 2023-11-28

**Authors:** Thomas Tscholl, Gösta Nachman, Bernhard Spangl, Ida Scalmani, Andreas Walzer

**Affiliations:** ^1^ Department of Crop Sciences, Institute of Plant Protection University of Natural Resources and Life Sciences, Vienna Vienna Austria; ^2^ Department of Biology University of Copenhagen Copenhagen Ø Denmark; ^3^ Department of Landscape, Spatial and Infrastructure Sciences, Institute of Statistics University of Natural Resources and Life Sciences, Vienna Vienna Austria

**Keywords:** biological control, climate change, intergenerational plasticity, Phytoseiidae, predator–prey interactions, Tetranychidae

## Abstract

The more frequent and intense occurrence of heat waves is a challenge for arthropods because their unpredictable incidence requires fast adaptations by the exposed individuals. Phenotypic plasticity within and across generations might be a solution to cope with the detrimental effects of heat waves, especially for fast‐developing, small arthropods with limited dispersal abilities. Therefore, we studied whether severe heat may affect the reproduction of a pest species, the spider mite *Tetranychus urticae*, and its counterpart, the predatory mite *Phytoseiulus persimilis*. Single offspring females with different parental thermal origins (reared under mild or extreme heat waves) of both species were exposed to mild or extreme heat waves on bean leaves over 10 days, and the oviposition, egg sizes, survival, and escape behavior of the females were evaluated daily. The total losses of predators mainly via escapers were very high compared to prey, which makes a separation between selective and plastic effects on shifted reproductive traits impossible. Predator females laid smaller eggs, while their consumption and oviposition rates were unaffected during extreme heat waves. In comparison, larger prey females fed more and produced more, but smaller, eggs due to within‐ and trans‐generational effects. These advantages for the prey in comparison to its predator when exposed to extreme heat waves during the reproductive phase support the trophic sensitivity hypothesis: higher trophic levels (i.e., the predator) are more sensitive to thermal stress than lower trophic levels (i.e., the prey). Furthermore, the species‐specific responses may reflect their lifestyles. The proactive and mobile predator should be selected for behavioral thermoregulation under heat waves via spatiotemporal avoidance of heat‐exposed locations rather than relying on physiological adaptations in contrast to the more sessile prey. Whether these findings also influence predator–prey interactions and their population dynamics under heat waves remains an open question.

## Graphical abstract

The more frequent and intense occurrence of heat waves affects many aspects of an arthropod's life, which may disturb or enhance interactions with other species, such as between a predator and its prey. Here, we assessed these effects on the reproductive output of a pest mite, *Tetranychus urticae*, and its natural enemy, the predatory mite, *Phytoseiulus persimilis*. Our results suggest advantages for the prey in comparison to its predator when exposed to extreme heat waves during the reproductive phase.

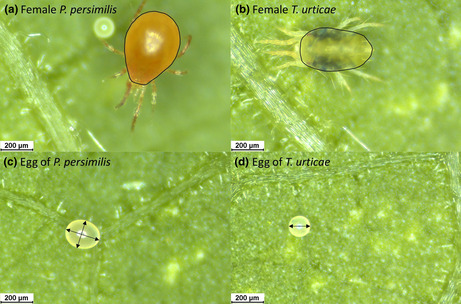

## 1 INTRODUCTION

Ongoing climate warming is resulting in heat waves that last longer, occur more frequently, and reach higher maximum temperatures (Meehl & Tebaldi, 2004). This constitutes a special challenge for ectotherms because of their function on ambient temperatures (Kingsolver et al., 2013; Ma et al., 2021; Vasseur et al., 2014). Mainly, the upper thermal limits of survival are used as predictors to determine the heat sensitivity of ectotherms (Geerts et al., 2015; Kellermann et al., 2012). However, the appropriate survival limits of ectotherms do not necessarily imply that their population growth and persistence are ensured under heat stress, because other traits may often be more heat‐sensitive (Walsh et al., 2019). Thus, the thermal limits of reproduction may serve as an indicator of how extreme temperatures impact a species, in particular whether, in the long run, heat waves are likely to be harmful to an endangered population (Costa et al., 2023).

Which strategies are likely to be accomplished by ectothermic females during the reproductive phase under heat waves? First, in species with finite growth, females experiencing heat waves may simply divert all energy from reproduction to the maintenance of their basic body functions and reinvest in reproduction under suitable thermal conditions (Roff, 1992). Such a strategy is accomplished by the heat‐sensitive yellow dung fly *Scathophaga stercoraria*, which enters reproductive quiescence in hot summers to restart reproduction in the cooler autumn periods (Blanckenhorn et al., 2001). Second, based on life history theory, heat stress may directly induce flexible shifts in egg number and/or egg size in reproducing females in order to maximize their own lifetime reproductive output (Roff, 1992; Stearns, 1992). Commonly, more of the finite energy for reproduction is allocated to the number of eggs, but not their size, when females are exposed to heat stress [e.g., geckos (Starostová et al., 2012), mites (Tscholl et al., 2023)]. A potential cause could be that high temperatures result in faster juvenile growth and earlier adulthood, thereby increasing juvenile survival and population growth. Consequently, selection should favor smaller egg sizes, which also enables females to invest more in the number of eggs (Sibly & Calow, 1983; Taylor & Williams, 1984; Yampolsky & Scheiner, 1996). Deviations from this trend are documented for crickets [higher number and larger size of eggs (Stahlschmidt et al., 2022)], lizards [higher egg number but insensitive egg sizes (Hall & Warner, 2017)], butterflies [lower egg number and smaller size (Janowitz & Fischer, 2011)] and beetles [both egg number and size are insensitive (Vasudeva, 2023)]. However, temperature also indirectly influences egg size and egg number through the maternal phenotype (Angilletta Jr, 2009). The capacity of females to acquire resources for reproduction greatly affects their ability to invest in egg number and/or size, with the clear advantages of large over small females (Angilletta, 2006; Parker & Begon, 1986; Roff, 1992). Additionally, large females should be able to produce egg sizes that are beyond the capacity of small females simply because of morphological constraints (Roitberg et al., 2013; Walzer & Schausberger, 2013). Nonetheless, most ectothermic females reach adulthood at a smaller size under high temperatures (Angilletta Jr, 2009; Atkinson, 1994), but there are also exceptions to this temperature–size rule (Fu et al., 2016; Kingsolver et al., 2007; Walters & Hassall, 2006; Walzer et al., 2020). Third, in species with sexually dimorphic egg sizes, heat‐stressed females may also invest energy depending on offspring sex, as documented in predatory mites exposed to food stress (Walzer & Schausberger, 2015) and seed beetles exposed to pesticides (Castano‐Sanz et al., 2022).

Another aspect concerns the mechanisms generating shifts in egg number and size under heat stress. It is commonly recognized that both genetic adaptation and plasticity could contribute to climate change responses (Merilä & Hendry, 2014), but the sudden occurrence of heat waves requires a fast response, favoring plasticity (Harmon & Pfennig, 2021; Walzer et al., 2020). Such modifications can appear within a generation, termed within‐generational plasticity (WGP), but parents can also induce shifts in offspring traits without changing the genetic code, which is termed trans‐generational plasticity (TGP) (Bonduriansky et al., 2012; Donelson et al., 2018). TGP should be favored in fast‐developing ectotherms with limited dispersal abilities (Walzer et al., 2020). First, short generation times increase the likelihood of a positive autocorrelation between parental and offspring environments, making the parental heat experience a reliable predictor of the thermal conditions of their offspring (Burgess & Marshall, 2011). Second, dispersal‐limited ectotherms are forced to remain in heat‐exposed sites, which makes plastic modifications more expectable. This applies, for instance, to aphids, spider mites, and predatory mites, and TGP induced by high temperatures is documented in some of these taxa (Jeffs & Leather, 2014; Margolies & Wrensch, 1996; Özder, 2002; Walzer et al., 2020). However, it is still an ongoing debate to what extent genetic modifications and/or plasticity can contribute to the reproduction of the ectotherms under heat stress (Walsh et al., 2019).

In this paper, we evaluate the reproductive performance of the predator *Phytoseiulus persimilis* Athias‐Henriot (Acari: *Phytoseiidae*) and its herbivorous prey, *Tetranychus urticae* Koch (Acari: *Tetranychidae*), under heat wave conditions. The predator is specialized to feed on spider mites and is used as a very successful natural enemy in several crop systems, both in the field and in greenhouses, to control *T. urticae* (Mcmurtry & Croft, 1997). Both mite species are characterized as income breeders with fast juvenile development (4–10 days), small adult body sizes (300–500 μm body length), sexual size dimorphism (smaller males), finite growth (Crooker, 1985; Sabelis, 1985b), and high reproductive output (up to 80 eggs/female predator and 160 eggs/female prey) under optimal conditions (Lü et al., 2019; Tscholl et al., 2022, 2023; Walzer et al., 2022; Wrensch & Young, 1975). A single mating is sufficient for life‐time reproduction of both species (Lü et al., 2019; Walzer & Schausberger, 2014), which can last up to 30 days (Li & Zhang, 2018; Walzer & Schausberger, 2014). Female offspring are numerically dominant under optimal conditions, but the sex‐ratio may shift to become more equal or even male‐biased under harsh conditions (Margolies & Wrensch, 1996; Tscholl et al., 2023; Walzer & Schausberger, 2014, 2015). Both species inhabit mainly the top parts of plants, where they are exposed to fluctuating daily and seasonal environmental conditions (Walzer et al., 2022). Their active dispersal abilities are restricted to ambulant movement, with the predator being more mobile than its prey (Gerson, 1985; Skirvin & Fenlon, 2003).

The predator is superior over prey in the capacity for population increase under moderate summer conditions (Sabelis, 1985a), which usually leads to a rapid suppression of the spider mite populations by the predator (e.g., Schausberger & Walzer, 2001; Walzer et al., 2009). Divergent results can be obtained from studies evaluating the control success of *P. persimilis* on spider mites under higher temperatures. The predator failed to control spider mites under hot and dry conditions (constant +27°C, 40% RH) in a climate chamber (Stenseth, 1979), whereas spider mite populations declined rapidly under heat wave conditions by the use of the predator in clementine orchards (Urbaneja‐Bernat et al., 2019). Nonetheless, the authors of the latter study also mentioned that spider mite outbreaks are likely to be more frequent because of ongoing climate warming.

Laboratory studies indicate that reproductive prey females are less heat‐sensitive than predatory mites (Bayu et al., 2017; Rojas et al., 2013; Urbaneja‐Bernat & Jaques, 2021). The reproduction of prey (*T. urticae*) peaked around 35°C when exposed to a temperature gradient from 10 to 40°C. The predator (*P. persimilis*), however, deposited the highest number of eggs at 25°C and 30°C, whereas egg production at 35°C was negligible (Urbaneja‐Bernat & Jaques, 2021). In contrast, these species‐specific differences were less pronounced under heat wave conditions. Exposed to mild (daily maximum temperature = 32°C) and extreme (daily maximum temperature = 38°C) heat wave conditions, both species increased their reproductive output at the cost of smaller eggs. Juvenile thermal acclimation extenuated this effect on egg sizes of prey, whereas the opposite was the case in female predator eggs (Tscholl et al., 2023). However, the potential effects of heat wave‐experienced parents on the reproductive output of their offspring under heat waves have not yet been evaluated for *P. persimilis* and *T. urticae*.

Consequently, we exposed prey and predator offspring females, deriving from heat wave‐exposed parents to mild heat waves (representative for present climatic conditions), and extreme heat waves (representative for prospective climatic conditions) to record their body size, escape behavior, adult survival, number of eggs produced per day, and egg size. We hypothesize that (1) the mobile predator suffers more from total losses (escapers + dead females), mainly due to higher escape rates compared to the more sessile prey; (2) both species invest in reproduction also under extreme heat waves; (3) the consumption rates, and consequently also the oviposition rates, increase under extreme heat waves in both species; (4) higher egg numbers result in lower egg sizes; (5) heat wave‐experienced parents contribute to offspring reproductive output via direct shifts in consumption, egg sizes and number and/or indirect shifts in adult body size of offspring; and (6) in general, the prey gains higher benefits in relation to reproduction under extreme heat waves than the predator.

## 2 MATERIALS AND METHODS

### 2.1 Species origin and rearing

The laboratory populations of *P. persimilis* and *T. urticae* were founded by specimens obtained from a commercial producer (Biohelp, Vienna, Austria). Predators and prey were reared in an incubator [25 ± 1°C, 60 ± 10% RH, 16:8 (L:D) h] and a climate chamber [25 ± 2°C, 60 ± 15% RH, 16:8 (L:D) h], respectively. The predators were reared on arenas consisting of plastic tiles on foam cubes in plastic boxes (20 × 20 × 6 cm), which were half‐filled with water. Bean leaves infested with spider mites were added three times per week. The prey, *T. urticae*, was reared on whole bean plants (*Phaseolus vulgaris* L.).

### 2.2 Heat wave conditions

In the eastern parts of Austria, the predator *P. persimilis* is extensively applied to vegetables and soft fruits to control spider mites, so we focused on this economically important area to study the potential outcome of global warming. To determine the temperature values for mimicking mild (M) and extreme (E) heat waves, we analyzed the characteristics (frequency, intensity, and duration) of heat waves during 2011–2020 based on the daily minimum (*T*_min_) and maximum temperature (*T*_max_) values. These data were obtained from a meteorological station in the town of Eisenstadt (48.117° N, 16.567° E), capital of Burgenland, Austria (representative of Eastern Austria). Consecutive hot days were characterized as heat waves when the daily *T*_max_ values reached ≥30°C for the first three consecutive days; the daily *T*_max_ was below 30°C, but not 25°C for single days during a heat wave period; and finally, the average *T*_max_ was >30°C for the entire heat wave period (Huth et al., 2000; Kyselý et al., 2000). The analyses revealed that the daily maximum temperatures did not exceed 35°C (85.95%) for the majority of heat waves during this time period. Thus, we created mild and extreme heat waves with the following temperature (*T*) and relative humidity (RH) values: (1) mild heat waves: *T*_mean_ = 22.6°C, *T*_max_ = 32.0°C, *T*_min_ = 16°C, RH_mean_ = 67.9%, RH_max_ = 85.0%, RH_min_ = 50.0%; (2) extreme heat waves: *T*_mean_ = 28.6°C; *T*_max_ = 38.0°C, *T*_min_ = 22.0°C; RH_mean_ = 60.0%, RH_max_ = 75.0%, RH_min_ = 50.0%. The photoperiod corresponded to long‐day conditions (L:D = 16 h:8 h). Mild and extreme heat waves are considered to represent heat wave conditions induced by present and prospective climate warming, respectively [see for details: Table A1 and Tscholl et al., 2022].

### 2.3 (Pre‐) experimental units, generating F1 females, and experimental procedure

To found the parental generation (F0), 15–20 females of either *P. persimilis* or *T. urticae* females from the rearing units were placed on detached bean leaves, which were positioned upside‐down on water‐saturated foam cubes in plastic boxes (20 × 20 × 6 cm), half‐filled with water. The predators were also supplied with spider mites. The females were allowed to deposit eggs for 6 h and were then removed (mean egg age: ±3 h). These rearing units were then exposed to either mild (M) or extreme (E) heat wave conditions during the juvenile development of the F0 generation. After reaching adulthood, the young parental females (F0) of *P. persimilis* or *T. urticae* were placed in cohorts of 15–20 females on detached bean leaves (identical juvenile rearing units as for the F0 generation), from which they were removed after 6 h. The deposited eggs (F1) were exposed to either mild or extreme heat wave conditions until the F1 females reached adulthood. These females were then placed singly in lockable cages [described in detail in Schausberger, 1997, Walzer et al., 2020] for 24 h under constant climatic conditions of 25 ± 1°C, 60 ± 10% RH, 16:8 h (L:D) to check fertility and standardize the hunger level. For the experiments, exclusively gravid offspring females (F1) were randomly selected and placed singly on the experimental units consisting of detached bean leaves (4.0 × 4.0 cm) on water‐saturated foam cubes in plastic boxes [detailed description of the experimental units in Tscholl et al., 2022]. Before an experiment started, the females were individually photographed using a digital microscope system (DMS 1000; Leica GmbH, Germany).

**FIGURE A1:**
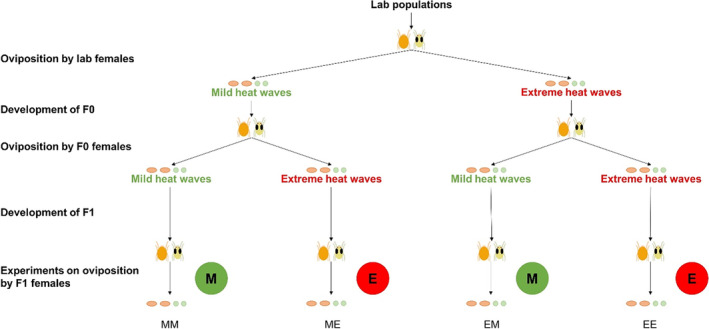
A schematic overview of the experimental procedure. Females and eggs of both mite species are depicted here for *P. persimilis* as 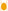 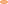 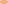 and for *T. urticae* as 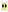 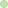 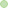. First, females from laboratory populations of both species were used to produce eggs (F0 generation), which were then raised to adults either under mild (M) or extreme (E) heat waves. When F0 individuals became adults, they produced F1 eggs, which were exposed to either mild (M) or extreme (E) heat waves during their juvenile development, thereby creating four groups of mites per species with different thermal histories (MM, ME, EM, and EE – first and second upper case letters indicate the parental and offspring heat wave conditions, respectively). Adult F1 individuals were transferred to experimental arenas, where they were exposed for 10 successive days to the same heat wave conditions as they experienced during juvenile development.

The F1 females were exposed to the same heat wave conditions as they experienced during their juvenile development for 10 days. This resulted in a full factorial design (for a schematic overview, see Figure A1) with the following treatment combinations: (1) parents (F0) and offspring (F1) developed under extreme heat waves [EE (first and second upper case letters indicate the parental and offspring heat wave conditions, respectively)]; (2) parents developed under extreme heat waves, offspring under mild heat waves (EM); (3) parents and offspring developed under mild heat waves (MM); and (4) parents developed under mild heat waves, offspring under extreme heat waves (ME). Predator females were provided with 150 spider mite eggs, which were replenished every third day. The state of the females (alive, escaped, dead), consumption (only for the predator), and the number of deposited eggs were recorded daily. Damage caused by the female spider mites is indicated by chlorotic spots on the leaves (Tomczyk & Kropczynska, 1985) and was determined at the end of the experiments by the evaluation of the size of the feeding scars using a transparent mm^2^ plastic screen. All deposited eggs were counted daily and thereafter destroyed except for two randomly chosen eggs, which were used for measurement of egg size and determination of sex. First, the sampled eggs were photographed individually with a digital microscope system (DMS 1000; Leica GmbH, Germany). Then, the eggs were placed individually on the sex‐determination units [similar to an experimental unit but with a smaller detached bean leaf (2.0 × 2.0 cm)] and reared to adulthood. Juvenile predators were provided with spider mites. The adult mites were finally sexed based on their sex‐specific body size and shape.

**FIGURE 1:**
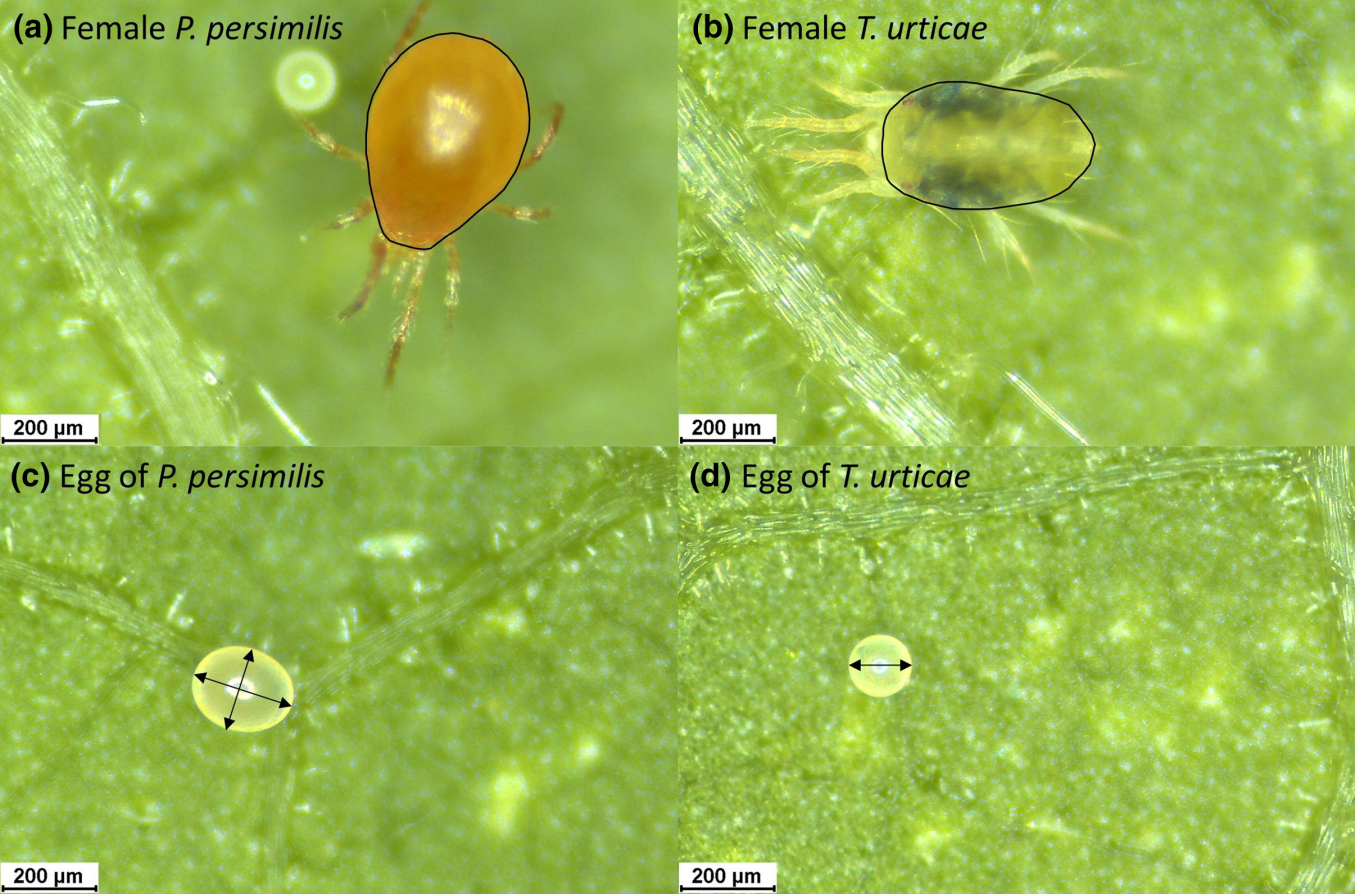
Size measurements of the perimeter of the idiosoma of adult female *P. persimilis* (a) and *T. urticae* (b), as well as the length and width of *P. persimilis* eggs (c) and the diameter of *T. urticae* eggs (d).

After finishing the experimental series, the perimeter of the female idiosoma was measured from the digital photos (Figure 1a,b). Additionally, the photos of the eggs were used to assess the volume of an egg by measuring (in μm) the semi‐major axis (*a*) and semi‐minor axis (*b*) (in the case of an ellipsoid predator egg) or its radius (*r*) (in the case of a globular prey egg) [for details, see Walzer et al., 2022, Figure 1c, d]. Subsequently, the volume (in μm^3^) of an egg was calculated from either $V=\frac{4}{3}\mathrm{\pi a}b^{2}$ (predator eggs) or $V=\frac{4}{3}\;\pi r^{3}$ (prey eggs). We used the software Leica Application Suite X (LAS X; Leica GmbH, Germany, Figure 1) for measurements of both eggs and adult females.

### 2.4 Statistical analyses

All statistical analyses were conducted by means of SAS OnDemand (SAS Institute 2021).

#### 2.4.1 Effects of heat waves on total losses induced by escaping and mortality

The purpose of this analysis was to investigate how adult F1 females of predators and prey responded (died, escaped) to the temperature regime experienced during the juvenile stage and/or the conditions experienced by their parents. The analyses of losses were divided into three separate analyses:

(i) *Total losses*: The total losses of F1 females (died, escaped) were compared between the two species, using a χ^2^‐test (PROC FREQ).

(ii) *Heat wave conditions*: A saturated log‐linear model was applied to analyze the primary effects of species (predator, prey), the parental heat wave conditions (F0: mild heat waves, extreme heat waves), the offspring heat wave conditions (F1: mild heat waves, extreme heat waves), as well as all interactions among these factors, on the binomially distributed response variable (*y*) of the offspring females (survived/died; remained/escaped). Data were modeled as

(1) $$y=\beta_{0}+\beta_{1}F0+\beta_{2}F1+\beta_{3}F0∙F1+ɛ$$

where F0 and F1 are two classification (dummy) variables defining the heat wave conditions experienced by the parents (the F0 generation) and their offspring (the F1 generation). Thus, both F0 and F1 could take two values: M = mild heat wave conditions and E = extreme heat wave conditions. A significant $\beta_{1}$ is interpreted as a potential TGE (trans‐generational effect), a significant $\beta_{2}$as a potential WGP (within‐generational plastic effect), and a significant $\beta_{3}$ as a potential TGP (trans‐generational plastic effect) [for details, see Walzer et al., 2022]. Based on the likelihood ratio, non‐significant terms were stepwise removed from the saturated model. Calculations were carried out by means of PROC CATMOD in SAS.

(iii) *Age‐dependent escape and survival rates:* PROC LIFETEST in SAS was used to generate the escape and survival functions (i.e. the proportion of mites that have not escaped or died at day *t*) of both species based on Kaplan–Meier's estimators and to estimate their hazard functions (probability of escaping or dying at a given age) using Epanechnikov smoothed‐kernel functions. Log‐rank tests were used for testing differences between the species. The number of non‐escaping or surviving individuals at day 0 was used as the reference point for escape or survival probabilities, i.e. *f*(*t* = 0) = 1. Females that died (in the case of escape functions) or escaped (in the case of survival functions) during an experiment were removed from the analysis. Data for the heat wave combinations were pooled. Additionally, we investigated the influence of heat wave conditions on escape risk and survival probability by stratifying data into four strata corresponding to the four treatment groups (MM, ME, EM, and EE) or by stratifying according to parental treatment (F0) (i.e., MM and ME vs. EM and EE) and offspring treatment (F1) (i.e., MM and EM vs. ME and EE) for each species separately.

#### 2.4.2 Effects of heat waves on consumption, egg number, and egg size

The purpose of these analyses was to investigate how food consumption, oviposition rate, and egg size of F1 females of both species were affected by temperature conditions during the juvenile stage and the temperature conditions experienced by their parents. We also aimed at investigating how these traits varied with the females' age.

(i) Total Consumption: The total consumption rate per female during the experimental period (10 days) was analyzed for each species separately. Equation 1, representing a two‐way ANOVA with interaction, was fitted to the data by means of PROC GLM, assuming that the residuals *ε* are normally distributed with a zero mean. This assumption was checked by means of Q‐Q plots (PROC UNIVARIATE). Female body size was included as a covariate in the model since large females, all other things being equal, are likely to consume more prey than small females. Females that either died or escaped during an experiment were removed from the analysis.

(ii) *Age‐dependent predation rates*: Time trends in the daily consumption rates over a period of 10 days were only analyzed for the predator. Since the females used in the experiments eclosed over a period of 1 day, their exact age was unknown. Consequently, it was assumed that their average age during the first day of an experiment was 3.5 days and 12.5 days during the last day. The predation rate at age *t* (denoted *P*(*t*)) was modeled by means of a generic non‐linear model (see Tscholl et al., 2023) given as

(2) $$y\left(t\right)=at^{b}e^{\mathrm{ct}+dt^{2}}$$

where *y*(*t*) denotes a given trait value at age *t* (e.g. *y*(*t*) = *P*(*t*)).

Equation 2 describes biological phenomena where the dependent variable (*y*(*t*)) cannot take negative values (e.g., feeding, oviposition, survival, or emigration) and where the dependent variable may either increase or decrease monotonically with age or reach a maximum at intermediate age, depending on the values of the equation's four shape parameters (*a*, *b*, *c*, and *d*). *b* determines whether *y*(*t*) at age *t* = 0 is either 0 (if *b* > 0) or positive (if *b* = 0).

Since newly eclosed predator females (at age *t* = 0) are capable of feeding, *b* in equation 2 is set to 0. Thus, *a* corresponds to the predation rate of newly eclosed females, while *c* and *d* express how aging affects the predation rate. If *c* > 0 and *d* < 0, it indicates that the predation rate peaks at age $t=\frac{-c}{2d}$. The parameters of equation 2 were estimated by fitting the model to the data by means of PROC NLIN in SAS.

Equation 2 was linearized to model ln *P*(*t*) as

(3) $$\ln\;P\left(t\right)=\beta_{0}+\beta_{1}t+\beta_{2}t^{2}+\varepsilon$$

where $\beta_{0}$ corresponds to ln *a*, $\beta_{1}$ to *c*, and $\beta_{2}$ to *d*. *ε* denotes the residual, which is assumed to be normally distributed.

Since daily consumption rates of females were followed over 10 successive days, each individual contributed with several observations. Therefore, we analyzed data by means of a repeated measurement analysis, using a female's ID nested within each treatment combination (MM, ME, EM, and EE) as the repeated factor. We used PROC GEE (generalized estimating equations) to estimate the parameters of the model. When a female died or escaped during an experiment, consumption rates on the following days were treated as missing values. The full model included the heat wave condition of the females (F1) and their parents (F0) as classification variables and female age (*t*) as a covariate, as well as the interactions between the three factors. The parameters associated with F0, F1, and F0*F1 were used for testing the significance of potential TGE, WGP, and TGP effects (see total losses), respectively. Since *P*(*t*) could take zero values, one was added to the observed predation rates prior to the ln‐transformation, which also served to stabilize the variance. The underlying distribution of the dependent variable was considered to be approximately normal, i.e. *Dist* = normal and *Link* = identity. Non‐significant factors (i.e. *p* > .05) were stepwise excluded from the model based on Type 3 sums of squares (SS). Q–Q plots (PROC UNIVARIATE) were used to check the assumption of normally distributed residuals.

(iii) *Total oviposition*: The total number of eggs produced per female during the experimental period (10 days) was analyzed for each species separately in the same way as the total consumption data.

(iv) *Age‐dependent oviposition rates*: Fecundity per female was analyzed in the same way as the consumption rates. The daily oviposition rates at age *t* (denoted *F*(*t*)) of both species were modeled by means of equation 2, but since the oviposition rate of newly eclosed females is 0, *b* > 0. Females were assumed to be 3.5 days old on the first day and 12.5 days old on the last day of the experiment. Equation 2 was linearized as

(4) $$\ln\;F\left(t\right)=\beta_{0}+\beta_{1}\ln t+\beta_{2}t+\beta_{3}t^{2}+\varepsilon$$

where $\beta_{0}$ corresponds to ln *a*, $\beta_{1}$ to *b*, $\beta_{2}$ to *c*, and $\beta_{3}$ to *d*. The effects of heat wave conditions were included in equation 4 by means of the two classification variables (F0 and F1), and their interaction (F0*F1) similar to equation 1. The full model also included the interactions between the independent variables. As with consumption rates, data were modeled by means of repeated measurement analysis using PROC GEE. In order to apply observations where *F*(*t*) = 0, one was added to all observations prior to the analysis. The two species were analyzed separately. Non‐significant terms were removed from the model as described for consumption rates.

(v) *Egg size*: The overall difference between the size of male and female eggs was analyzed by means of a one‐way ANOVA (using PROC GLM) with sex as the independent variable. Each species was analyzed separately. Furthermore, heat wave effects on egg sizes were analyzed by means of equation 1 using PROC GLM. The effect of the mother's body size was included in the analysis as a covariate. Pairwise comparisons between treatment combinations were conducted by means of Tukey's post‐hoc tests.

(vi) *Effect of female age on egg size*: The size of an egg produced by a female of age *t* [denoted *V*(*t*)] was predicted by means of equation 3 using *t*, *t* ^2^, *Sex*, F0, F1, as well as their second‐order interactions, as predictor variables. Because the same females contributed with several eggs during an experiment, we used the repeated measurement analysis factor (PROC GEE), similar to the analyses of consumption and oviposition rates, to analyze the ln‐transformed values of *V*(*t*).

#### 2.4.3 Determination of the selective effects of total losses on observed trait shifts

High total losses of the F1 females caused by the heat waves can lead to selection effects, which do not allow the separation of genetic from plastic responses to heat waves (Kielland et al., 2017). Therefore, we considered shifts in consumption, egg number, and size as plastic effects when the total losses were not affected by the heat waves and the majority of the F1 females (≥60%) remained on the detached bean leaves during the entire experimental period of 10 days.

#### 2.4.4 Effects of heat waves on the body size of F1 females

The purpose of this analysis was to investigate whether the body size of F1 adult females of predators and prey was affected by the temperature regime experienced during the juvenile stage and/or the temperature regime of their parents. Any shift in female body size of the F1 generation clearly indicates a plastic response to heat waves during their juvenile development because the juvenile mortality of predators and prey offspring is low and not affected by the heat wave conditions (Walzer et al., 2022).

The perimeter of the idiosoma, measured before exposing the females to heat waves during adulthood, was used as a proxy for body size. The total number of measured individuals (*n*) was 152 predators and 206 prey. Body size was analyzed for each species separately using equation 1 in the same way as the total consumption data.

Trait values are presented in the text as averages, followed by 95% confidence limits in brackets, and as bars, dots, and vertical lines in the figures.

## 3 RESULTS

### 3.1 Effects of heat waves on total losses induced by escaping and mortality

*(i) Total losses*: The statistical analysis revealed a highly significant species‐specific effect (PROC FREQ: $\chi_{2}^{2}$ = 147.9; *p* < .0001) on the total losses (escaped, died) of predator and prey females: 40 (26%) out of 155 predators (*P. persimilis*) and 135 (65%) out of 206 prey (*T. urticae*) remained alive on the bean leaves until day 10, respectively.

(ii) *Heat wave conditions*: F0 heat wave effects on total losses were non‐significant, and all terms containing F0 were removed from the log‐linear model. The reduced model consisted of three primary factors (species, F1, and response) and three interaction terms: species*response ($\chi_{2}^{2}$ = 104.24; *p* < .0001), F1*response ($\chi_{2}^{2}$ = 6.19; *p* = .0453), and species*F1*response ($\chi_{2}^{2}$ = 6.16; *p* = .0460). The F1 heat wave effects on total losses were dependent on the species. Thus, the number of remaining prey females on the bean leaves was not affected by F1 heat wave conditions (mild versus extreme heat waves: 65% vs. 66%), whereas more predators remained on the bean leaves under mild heat waves (29% vs. 23%).

(iii) *Age‐dependent escape and survival rates*: Pooled over heat wave conditions, the escape and survival functions for the predator and prey were found to be species‐specific (escape functions, log‐rank test: $\chi_{1}^{2}$ = 117.83; *p* < .0001; survival functions: $\chi_{1}^{2}$ = 5.980; *p* = .0145). 111 out of 151 predator females (74%) escaped from the detached bean leaves during the experimental period of 10 days, whereas the number of escaping prey females was much lower (21 out of 156: 13%). In contrast, few predator females died on the leaf surfaces during exposure to heat waves (4 out of 44: 9%) compared to prey females (50 out of 185: 27%). The Epanechnikov smoothed‐kernel hazard functions indicated that the escape probability for the predator and the risk of dying for both species peaked around 3 days after the start of an experiment, whereas the escape risk of the prey was highest in the beginning and then decreased monotonously with time.

**FIGURE 2:**
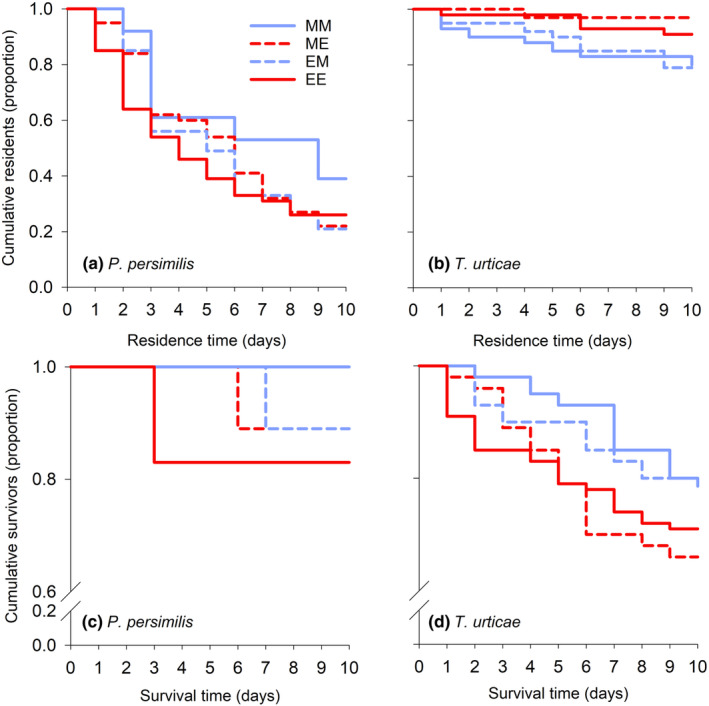
Heat wave effects on the escape (cumulative residents plotted against residence time, a,b) and survival (cumulative survivors plotted against survival time, c,d) functions of predator (a,c) and prey (b,d) F1 females. F1 females, originating from parents reared under mild (M) or extreme (E) heat waves, and F1 females exposed to mild (M) or extreme (E) heat waves are labeled by the first and second upper case letters.

The escape‐ and survival functions of the predator were neither influenced by the main factors F0 and F1, nor by their interactions (Table A2) (Figure 2a,c). The same applied to the prey except that the effect of F1 on escape probability was significant (Table A2), indicating that mild heat wave conditions increased the prey's tendency to move away (Figure 2b,d).

### 3.2 Heat wave effects on food consumption, egg number, and size

(i) *Total consumption*: Pooled over time, F0 and F1 heat wave conditions and female body size did not affect the feeding rates of the predator females (Table 1, see Table A3 for means). In contrast, food consumption of prey females was significantly influenced by both F0 and F1 heat wave conditions, but not by female body size and interaction F0*F1 (Table 1). Pooled over F1 heat wave conditions, consumption rates of prey females originating from parents exposed to mild heat waves were significantly higher (*t*_133_ = 3.29; *p* = .0013) compared with females derived from parents exposed to extreme heat waves [141.81 (133.03–150.59) mm^2^ vs. 120.81 (11.69–129.92) mm^2^]. Pooled over F0 heat wave conditions, extreme heat waves (*t*_133_ = 4.83; *p* < .0001) boosted the consumption activities of females by about 25.74% [114.84 (105.88–123.80) mm^2^ vs. 144.40 (136.07–152.73) mm^2^].

**TABLE 1:** Effects of parental (F0) and offspring (F1) heat waves (mild or extreme) on the body size of adult females as well as on their consumption, oviposition, and sex‐specific egg sizes, with the body size of *P. persimilis* and *T. urticae* included in equation 1 as a covariate.

Parameter	Source of variation	*P. persimilis* females				*T. urticae* females			
		$\hat{\beta }$	*t*	Df	*p*	$\hat{\beta }$	*T*	Df	*p*
Consumption	F0	0.0577	0.07	35	.9425	−32.7179	−3.90	130	**.0002**
	F1	0.9360	0.97	35	.3368	23.3193	2.76	130	**.0065**
	F0*F1	0.7380	0.63	35	.5332	23.4743	1.94	130	.0648
	Body size	0.0141	1.80	35	.0812	−0.0756	−1.54	130	.1260
Oviposition	F0	0.1872	1.45	35	.1417	−0.2568	−0.52	130	.6606
	F1	0.0767	0.51	35	.6140	4.5828	9.16	130	**<.0001**
	F0*F1	0.0828	0.45	35	.6556	−0.3538	−0.49	130	.6253
	Body size	0.0007	0.61	35	.5470	0.0061	2.07	130	**.0402**
Egg sizes (♂)	F0	0.0094	0.15	231	.8815	−0.0135	−1.16	1148	.2458
	F1	−0.3747	−5.29	231	**<.0001**	−0.0543	−4.96	1148	**<.0001**
	F0*F1	0.0570	0.71	231	.4796	−0.0012	−0.08	1148	.9381
	Body size	−0.0005	−0.69	231	.4902	0.0001	2.32	1148	**.0203**
Egg sizes (♀)	F0	−0.0444	−1.25	661	.2135	−0.0047	−0.47	1121	.6415
	F1	−0.4519	−10.66	661	**<.0001**	−0.0871	−8.68	1121	**<.0001**
	F0*F1	0.1593	3.09	661	**.0021**	−0.0028	−0.18	1121	.8544
	Body size	0.0007	1.78	661	.0758	−0.0001	−0.99	1121	.3205
Body size	F0	10.4625	1.39	148	.1671	−3.0577	−0.25	202	.8048
	F1	−58,1789	−7.63	148	**<.0001**	11.9708	0.97	202	.3338
	F0*F1	12.4299	1.17	148	.2450	66.5174	3.92	202	**<.0001**

*Note*: Data were pooled over time. *p*‐Values in bold are significant at the 5% level.

**FIGURE 3:**
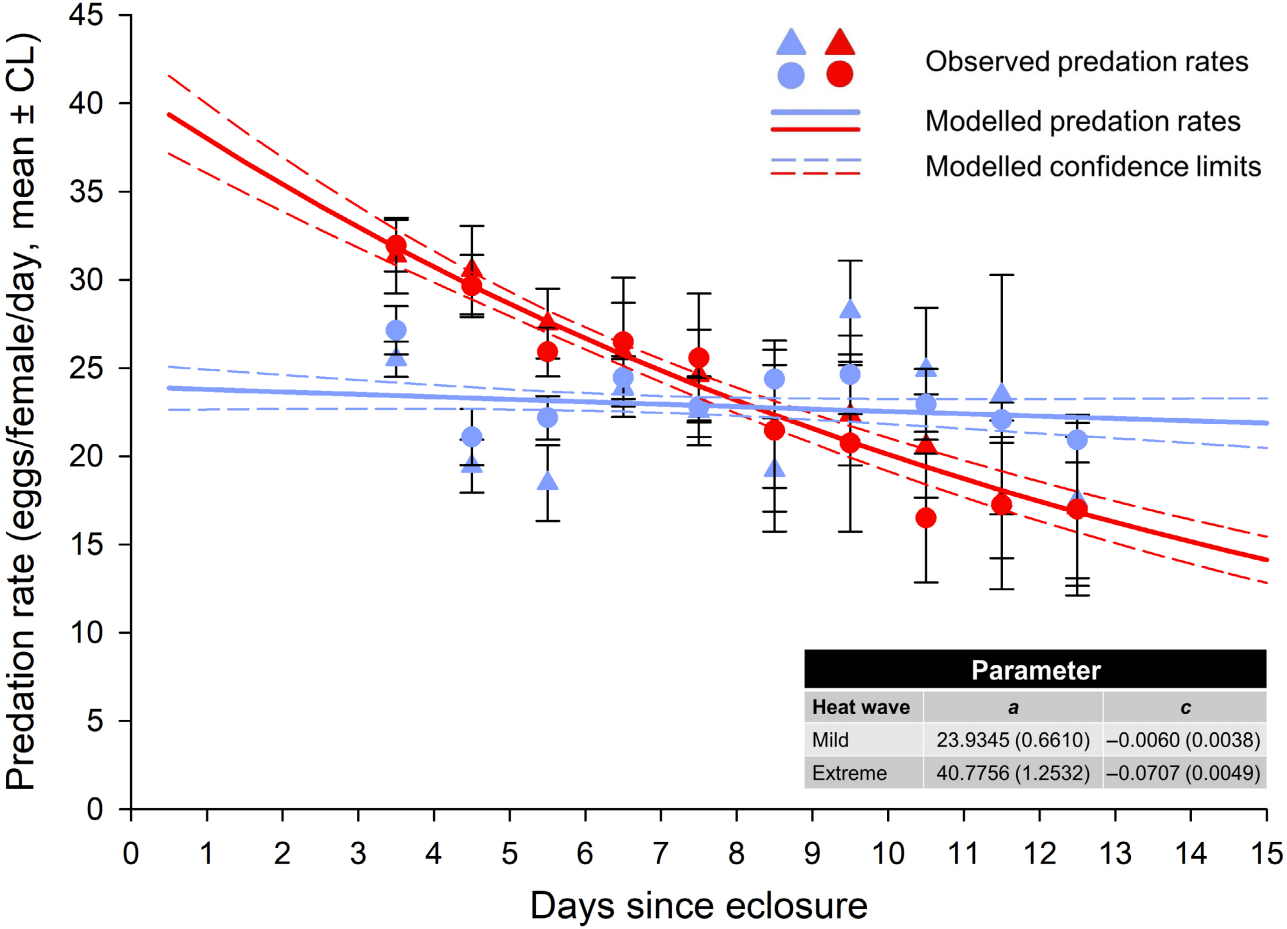
Daily predation rates of offspring (F1) of *P. persimilis* females [average ± 95% confidence limits (CL)] in function of the heat wave conditions (mild = blue, extreme = red) of F1 females and their parental (F0) origin. Triangles show F1 females derived from F0 females exposed to extreme heat waves, and dots mark F1 females derived from F0 females exposed to mild heat waves. Full lines show the predicted predation rates based on equation 2 with the 95% confidence limits (dashed lines). The estimated parameters (with the standard error in parentheses) for equation 2 are shown in the table.

(ii) *Age‐dependent predation rates*: The model explained 95.4% of the total variation in daily predation rates of females exposed to extreme heat wave and 95.7% of the variation when females were exposed to mild heat waves. Furthermore, the predation rates of F1 females exposed to mild heat waves were more or less independent of age, whereas the predation rates of heat‐stressed females declined monotonically with age. The two lines crossed circa 8 days after eclosure (Figure 3).

**FIGURE 4:**
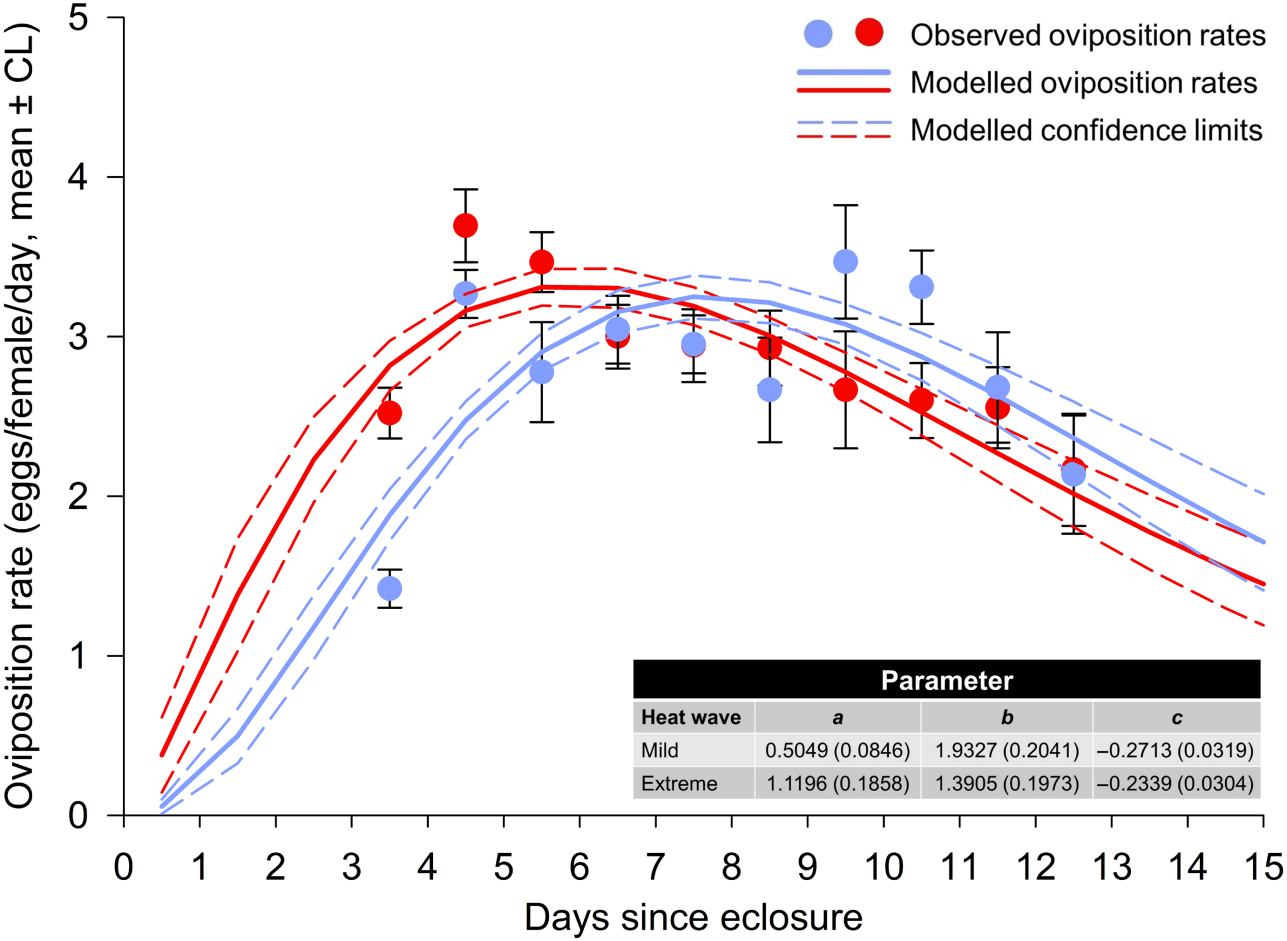
Daily oviposition rates of *P. persimilis* females exposed to extreme (red) or mild (blue) heat wave conditions pooled over parental (F0) heat wave conditions. Dots: average oviposition ±95% confidence limits (CL) for the mean. Lines: modeled oviposition (continuous lines) ± 95% confidence limits (dashed lines). The estimated parameters (with the standard error in parentheses) for equation 2 are shown in the table.

**FIGURE 5:**
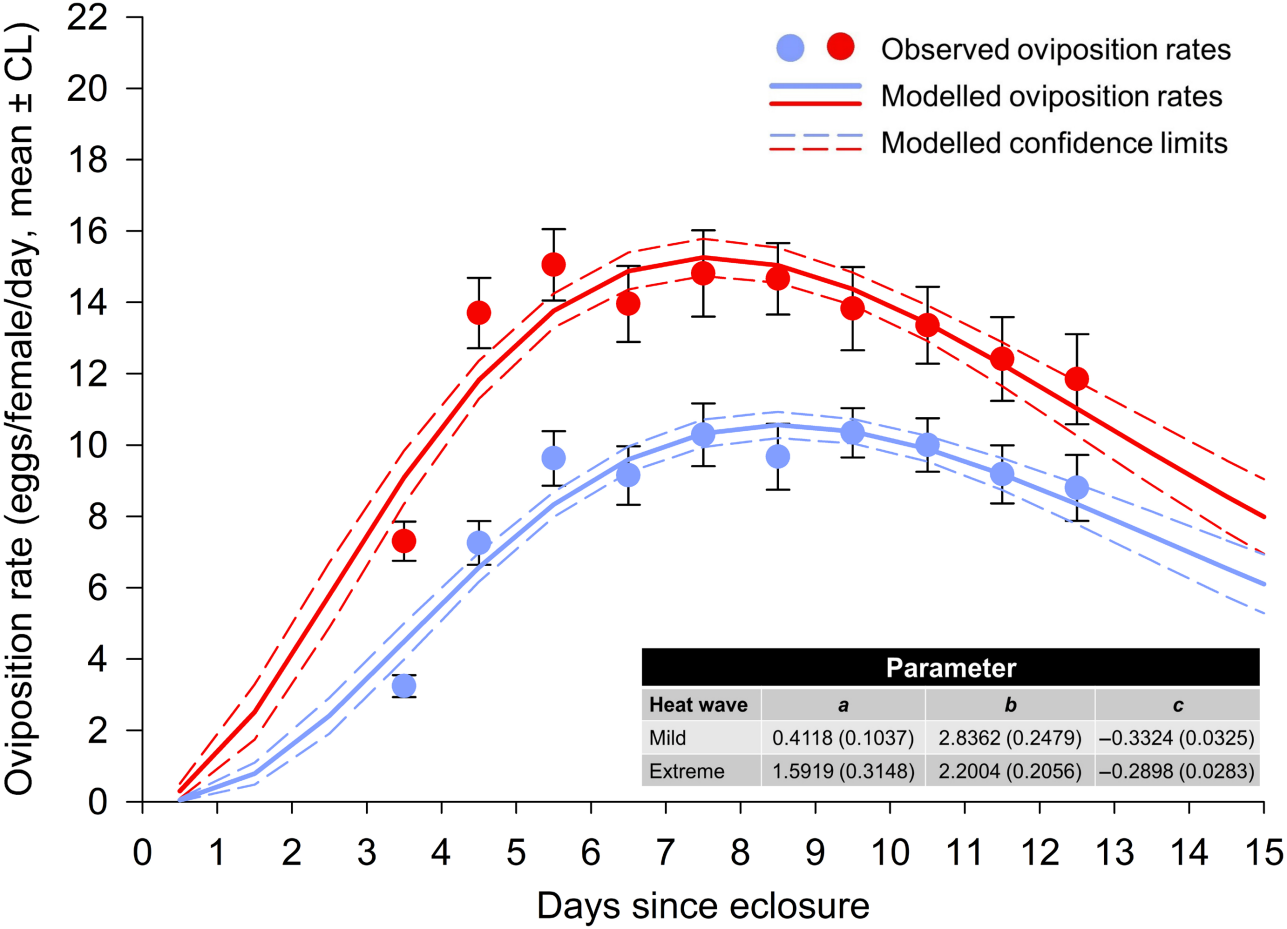
Daily oviposition rates of *T. urticae* females exposed to extreme (red) or mild (blue) heat wave conditions pooled over parental (F0) heat wave conditions. Dots: average oviposition ±95% confidence limits (CL) for the mean. Lines: modeled oviposition (continuous lines) ±95% confidence limits (dashed lines). The estimated parameters (with the standard error in parentheses) for equation 2 are shown in the table.

Both female age and heat wave conditions during F1 affected predation rates (*t*: $\chi_{1}^{2}$ = 40.40; *p* < .0001; F1: $\chi_{1}^{2}$ = 57.24; *p* < .0001), whereas there were no effects of heat waves conditions during F0. However, since the interaction between age and F1 was highly significant ($\chi_{1}^{2}$ = 31.08; *p* < .0001), extreme and mild heat wave conditions during F1 were modeled separately. Independently of F0 heat wave conditions, the predation rates of females exposed to extreme heat waves declined significantly with age ($\chi_{1}^{2}$ = 24.93; *p* < .0001), but not when the females were exposed to mild heat waves ($\chi_{1}^{2}$ = 2.27; *p* = .1319). Females exposed to mild heat waves consumed significantly more prey eggs ($\chi_{1}^{2}$ = 7.11; *p* = .0077) when F1 heat wave conditions corresponded to F0 conditions (Figure 3).

(iii) *Total oviposition*: Pooled over female age, neither female size, F0, nor F1 heat wave conditions influenced the average number of eggs deposited per day by predator females [MM: 2.65 eggs/female (2.53–2.78), ME: 2.94 (2.83–3.05), EM: 2.75 (2.59–2.90), and EE: 2.98 (2.85–3.12)] (Table 1). In contrast, the average daily fecundity of prey females was affected by their body size and F1 heat wave conditions (Table 1). Large prey females deposited more eggs, and exposure to extreme heat waves resulted in higher reproductive output [+33.77%, extreme versus mild heat waves: 13.00 (12.64–13.36) versus 8.61 (8.33–8.88) eggs/female].

(iv) *Age‐dependent oviposition rates*: The model explained 94.0% of the variation in the observed oviposition rates when the predator females had been exposed to extreme heat waves and 91.0% when the females experienced mild heat waves (Figure 4). The corresponding values for *T. urticae* were 87.5% and 87.2%, respectively (Figure 5).

The analysis of the within‐subject oviposition rates revealed that female age (*t*), In(*t*), and F1, but not F0, heat wave conditions significantly affected the egg production of both predator and prey. Additionally, the interaction terms F1*In(*t*) and F1*t were only significant for the predator (Table A4). The oviposition rates of the predator peaked earlier under extreme heat waves, but overall predator females produced almost the same number of eggs during the 10‐day period under mild and extreme heat waves [27.68 (26.54–28.82) versus 28.83 (27.15–30.52) eggs/female] (Figure 4). The number of eggs during the 10‐day period for the prey was 92.26 (87.35–97.18) eggs/female under mild heat waves versus 140.63 (135.40–145.85) eggs/female (*t*_133_ = 13.36; *p* < .0001) under extreme heat waves, demonstrating an increase in reproduction under extreme heat waves of 34.40%. Oviposition peaked 7–8 days after eclosure when heat waves were extreme and after 8–9 days when heat waves were mild (Figure 5).

(v) *Egg size*: In total, the volumes of 902 predator eggs (666 females and 236 males) produced by 143 females and 2279 prey eggs (1126 females and 1153 males) produced by 192 females were available for analysis.

Female predator eggs were significantly larger than male eggs [4.016 (3.986–4.045) ×10^6^ vs. 3.899 (3.840–3.937) × 10^6^ μm^3^ (*F*_1,900_ = 18.51; *p* < .0001)]. Along the same line, female prey eggs were significantly larger than male eggs [1.197 (1.190–1.205) ×10^6^ versus 1.140 (1.133–1.148) ×10^6^ μm^3^ (*F*_1,2277_ = 114.68; *p* < .0001)].

**FIGURE 6:**
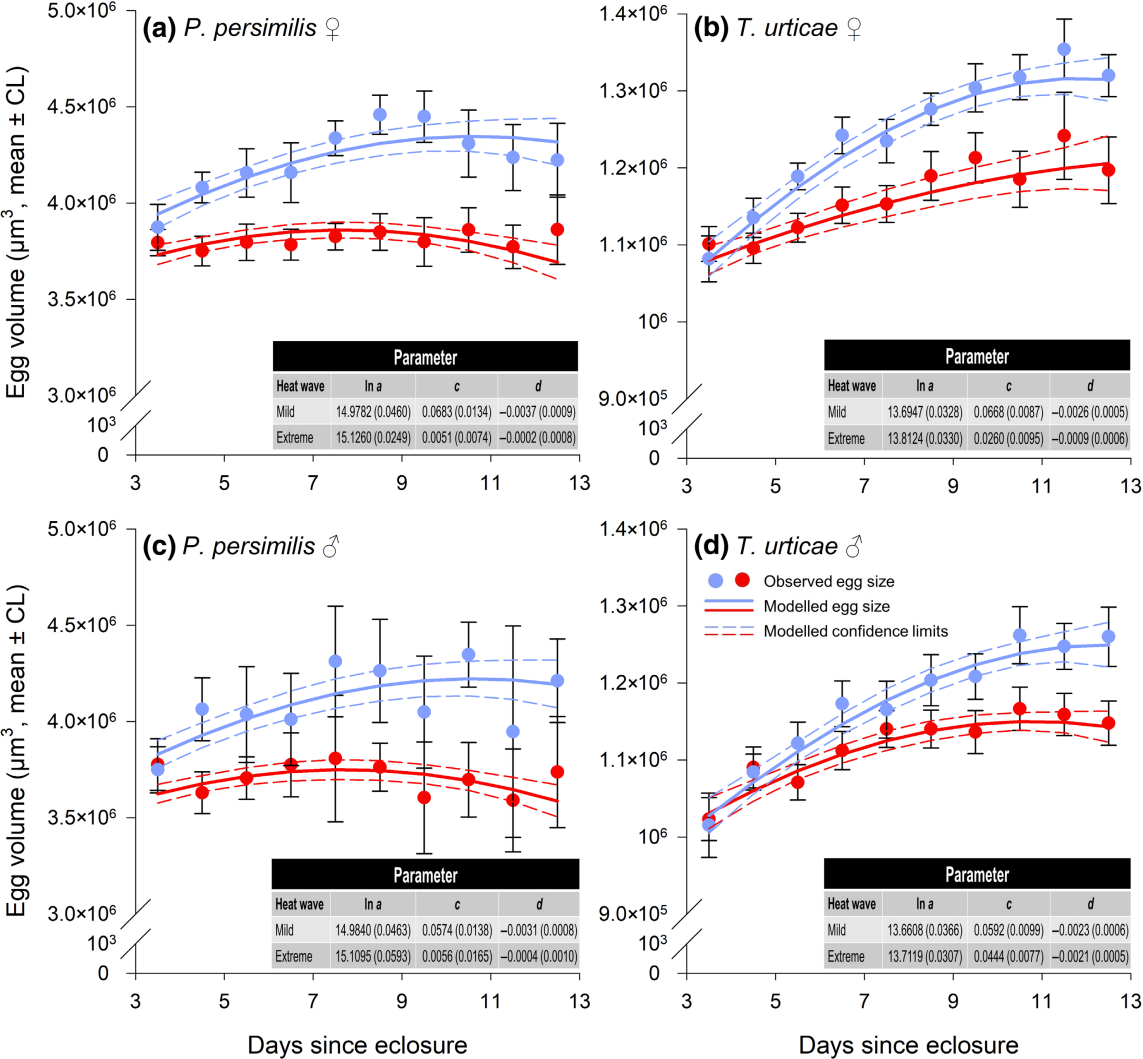
Offspring (F1) heat wave‐ and time effects on egg sizes of *P. persimilis* and *T. urticae*, pooled over parental (F0) heat waves. (a) female eggs of *P. persimilis*; (b) female eggs of *T. urticae*; (c) male eggs of *P. persimilis*; and (d) male eggs of *T. urticae*. Dots: average size ±95% confidence limits (CL) for the mean. Lines: modeled egg sizes (continuous lines) ±95% confidence limits (dashed lines). Blue: mild heat waves; Red: extreme heat waves. The relationship between female age (*t*) and ln‐transformed egg volumes (ln *V*(*t*)) was modeled by equation 3. The estimated parameters (with the standard error in parentheses) are shown in the tables.

Pooled over female age, female size, and F0 heat wave conditions did not affect the size of male and female predator eggs (Table 1). F1 effects resulted in smaller egg sizes under extreme heat waves. However, the size of female eggs was also affected by the interaction between F0 and F1 (Table 1). Thus, female eggs became relatively larger under extreme heat waves when the ovipositing females originated from parents, who also experienced extreme heat waves (Table A5). In contrast, the size of prey eggs was affected by F1 effects, leading to smaller eggs of both sexes under extreme heat waves (Table 1, Table A5). Female body size had a weak sex‐specific effect on egg sizes: large females tended to deposit relatively larger male eggs (Table 1).

(vi) *Effect of female age on egg size*: The GEE analysis revealed that all factors except F0 contributed significantly to predicting the ln‐transformed volumes of female and male eggs in the predator (Table A6). Overall, females exposed to mild heat waves laid the largest eggs. Furthermore, if heat wave conditions were mild, egg size tended to increase with female age, whereas a similar effect was not observed at extreme heat waves (Table A7) (Figure 6a,c).

Overall, eggs produced by young prey females were smaller than those produced by older females. However, heat wave conditions affected egg sizes differently, as the size of prey eggs increased with the mothers' age under mild heat waves but to a lesser extent under extreme heat waves. The difference was reflected by a highly significant interaction term between F1 and age (Table A6) (Figure 6b,d).

### 3.3 Effects of heat waves on the body size of F1 females

**FIGURE 7:**
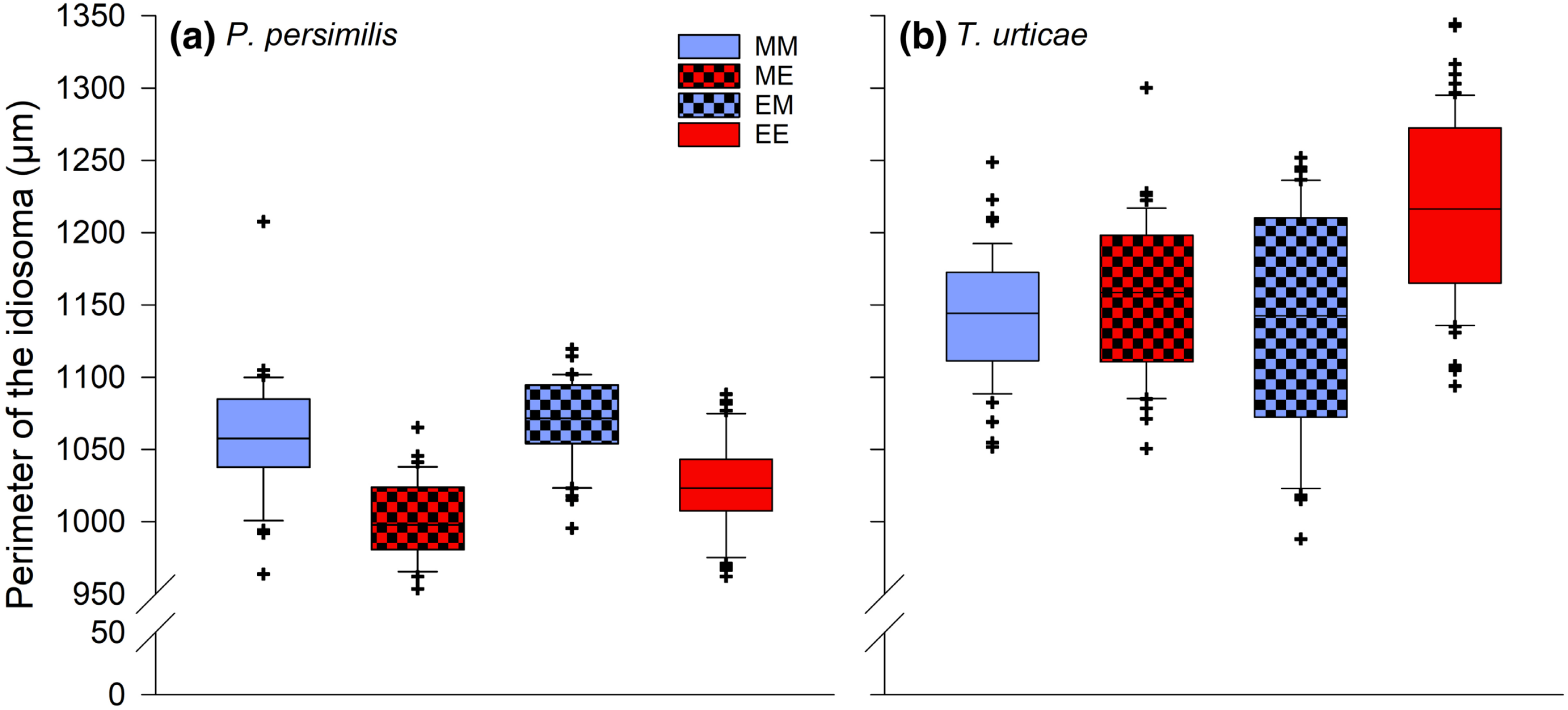
Shifts in body sizes (perimeter of the idiosoma) of the offspring (F1) females in function of their parental (F0) origin and heat wave conditions during juvenile development. F1 females, originating from parents reared under mild (M) or extreme (E) heat waves, and F1 females exposed to mild (M) or extreme (E) heat waves are labeled by the first and second upper case letters. The horizontal centerlines in each box represent the median, the box limits represent the interquartile (IQ) range from 25 to 75 percentiles, the whiskers extend the IQ range to 1.5 times, and outliers are depicted as symbols.

Body sizes of predator females were only affected by the F1 heat wave conditions experienced during juvenile development (Table 1). Females grown up under extreme heat waves were significantly smaller than females exposed to mild heat waves [1013.42 (1006.38–1020.49) μm vs. 1064.32 (1055.95–1072.69) μm] (Figure 7a). In contrast, only the interaction of parental and offspring heat waves during juvenile development was significant in prey females (Table 1). Different parental treatments resulted in larger body sizes only when both the offspring females and their parents were exposed to extreme heat waves (MM, ME, EM versus EE: *p* ≤ .0001). The body size of females, derived from parents developed under mild heat waves, was insensitive to heat wave conditions during their juvenile development (ME versus MM: *p* = .995) (Figure 7b).

## 4 DISCUSSION

In accordance with our hypotheses, total losses of prey were small compared with the predator due to the high escape rates of the latter. Both species did not divert all energy into the maintenance of their basic body functions but also invested in egg production under extreme heat waves. Consumption and oviposition rates of the prey increased under extreme heat waves, but not in the predator. The increased reproductive output of the prey resulted in smaller egg sizes. Direct parental environmental effects on the reproductive performance of their female offspring were marginal in both species and limited to shifts in female egg sizes (predator) and consumption (prey). Indirect parental environmental effects via body size shifts of F1 females were only observed in the prey: F1 females were larger when they and their parents grew up under extreme heat waves. These females also deposited more eggs and produced larger male eggs compared to females from parents that experienced mild heat waves. Finally, exposure to extreme heat waves resulted in smaller predator females, higher total losses mainly via escaping, decreasing consumption rates, and smaller deposited eggs, whereas the oviposition rates were unaffected by the intensification of mild to extreme heat waves. Prey females, however, were larger, fed more, and produced more but smaller eggs under extreme heat waves.

These results are in line with the trophic sensitivity hypothesis, which predicts that species at higher trophic levels (e.g., predators) should be more sensitive to thermal stress than species at lower trophic levels (e.g., their prey) (Cheng et al., 2016; Voigt et al., 2003). Large body size, low population densities, and high metabolic demands relative to prey are listed as potential causes as to why predators should be less physiologically adapted to heat stress than their prey (Baag & Mandal, 2023; Cheng et al., 2016; Voigt et al., 2003). Actually, the metabolic rates of *P. persimilis* as an actively hunting predator are higher than those of its prey (Bernstein, 1984), whereas the predator–prey body size ratio is in favor of the prey when adult females are compared (Tscholl et al., 2022; Walzer et al., 2022). Considering the high escape rates of *P. persimilis* females, it seems likely that the predator is selected for behavioral thermoregulation under heat stress rather than relying on physiological adaptations in contrast to the more sessile prey.

### 4.1 Reproductive strategies under heat waves

The consumption pattern nicely reflected the reproductive output of the two species, as higher energy gains obtained from increased consumption rates were converted into a higher fecundity of prey under extreme heat waves (+34.40%). Female predators exposed to extreme heat waves fed more when they were young but less when they grew older than those exposed to mild heat waves did, causing a concurrent decline in oviposition rates and, to a lesser extent, in egg size. However, averaged over 10 days, both predator groups laid almost the same number of eggs. This is in contrast to the performance of the parental generation of the predator, which had higher consumption rates and a 31.83% increase in egg numbers under extreme heat waves (Tscholl et al., 2023). It indicates that heat‐stressed females, whose mothers had also experienced either mild or extreme heat waves, responded by reduced investment in reproduction than their mothers did. Seemingly, extreme heat waves increased consumption rates in the short term, but since the energy costs associated with feeding activities showed a concurrent increase, a progressing energy deficit caused declining consumption rates, resulting in fewer and smaller eggs. Similar negative heat effects on fecundity were also reported for leaf miner flies and fruit flies (Huang et al., 2007), but their lower fecundities were traded off against increased heat tolerance because of the expression of heat shock proteins (Ozawa et al., 2012; Sørensen et al., 2003). However, it can only be hypothesized that the production of costly heat shock proteins, in combination with lower food intake, is also responsible for the reproductive performance of *P. persimilis* under extreme heat waves. In contrast, *T. urticae* benefited from a two‐fold indirect parental effect on the maternal phenotype: F1 females became larger during juvenile development under extreme heat waves when their parents were grown up under the same conditions. These larger females not only mitigated the otherwise negative effects of high temperatures on egg size by producing relatively larger male eggs but also by depositing more eggs under extreme heat waves. Consequently, large females not only seem to increase their own fitness under extreme heat waves by producing more eggs, but also the fitness of their male offspring by laying slightly larger eggs, a strategy rarely documented in the literature [e.g., Stahlschmidt et al., 2022].

### 4.2 Shifts in the reproductive performance induced by heat waves: Genetic modifications or plasticity?

Methodological shortcomings in experiments dealing with plastic effects may not allow for the separation of TGP from other sources of variation (Donelson et al., 2018; Kielland et al., 2017; Uller et al., 2013; Walzer et al., 2022). First, the partition of early‐offspring life‐stage conditions from the parental environment is difficult and hardly possible in some species. For instance, the complete offspring embryonic development of parthenogenetic water fleas [around 20% of the total juvenile development (Kielland et al., 2017)] takes place in the brood chambers of their mothers so that a plastic response of the F1 offspring might also be developmental plasticity (Donelson et al., 2018). In such cases, plasticity experiments should be conducted over two offspring generations. Our study subjects, the predatory mite *P. persimilis* and its preferred prey, the spider mite *T. urticae*, deposited their eggs on leaves, which were then removed from the maternal environment after 6 h (mean egg age of ±3 h). This short overlapping of the maternal and offspring environments constituted about 6.3% and 2.4% of the embryonal development and total juvenile development for the predator, respectively. The corresponding data for prey were 3.5% and 1.5%. Thus, the confounding effects of the parental environment on offspring responses should be small, although the offspring eggs spent some extra time in the maternal environment because of the egg formation inside their mothers before oviposition. A second aspect applies to selective losses via mortality and/or escaping in sexually reproducing species such as our study subjects. Plastic modifications cannot be distinguished from genetic effects when such losses are high, in particular if the losses interact with the offspring's environment (Donelson et al., 2018; Kielland et al., 2017). This was the case for the predator, because only one‐third (36%) of the predator females were still present on the detached bean leaves after 10 days. Additionally, the total losses of predator females were higher when they were exposed to extreme heat waves. In contrast, the number of prey females remaining on the leaves until the end of the experiments was high (65%) and not affected by heat waves. Consequently, a separation between selective and plastic effects on shifted traits is not possible in the predator. However, trait shifts in the prey induced by heat waves can most likely be attributed to plastic responses, although genetic effects cannot be completely excluded.

Another unexpected result was the marginal direct effects of parental heat wave exposure on the reproductive performance of their offspring when they reach adulthood. It seems to be a common finding that juvenile traits are more strongly influenced by the parental environment than fecundity traits in adults [reviewed in Herman & Sultan, 2011, Donelson et al., 2018, Yin et al., 2019]. Two non‐mutually exclusive causes may explain the observed marginal parental effects on offspring responses. First, juvenile development until adulthood under optimal conditions usually takes place on the natal sites chosen by the mothers in both mite species because of the limited dispersal abilities of their small juveniles (Kennedy & Smitley, 1985; Sabelis, 1985b). In contrast, young adults usually leave the natal sites to search for mates and suitable oviposition sites (Bitume et al., 2014; Nagelkerke et al., 1996). Moreover, the juvenile phase of both mite species is short relative to the subsequent reproductive period of the adult females (Bayu et al., 2017; Rojas et al., 2013). Taken together, the spatiotemporal correlation of parental and offspring environments is likely to be rather high for the juvenile period of offspring but then decrease in the subsequent adult phase of reproduction, thereby making the parental environment a less reliable predictor for the conditions experienced by their adult offspring. Second, plastic responses require that individuals detect and process cues occurring in their own environment, which may then trigger phenotypic changes. In contrast, trans‐generational modifications are the result of multi‐level processes by which parents convey information to their offspring. Subsequently, the information should be correctly translated in order to elicit an adequate change in the offspring's phenotype (Bell & Hellmann, 2019). Such complex communication mechanisms are more prone to errors compared to within‐generation information directly processed by individuals (Donelan et al., 2020). Consequently, in an evolutionary context, it may not pay to develop a costly mechanism with potentially high error rates as long as within‐generational plasticity (WGP) effects can lead to a similar outcome (Donelson et al., 2018). On the other hand, if the time factor is crucial for developing WGP effects, preadaptation to extreme conditions via TGP effects may provide fitness advantages.

### 4.3 Significance of female body size under heat waves

The body size of the experimental females was mainly determined by the parental (F0) and their own (F1) heat wave conditions during juvenile development, since both species have finite growth without further molting after maturity (Crooker, 1985; Sabelis, 1985b). The opposite shifts in female body sizes (smaller predator females and larger prey females under extreme heat waves) were plastic responses to heat stress because juvenile mortality of both F0 and F1 is low and rather insensitive to heat waves (Tscholl et al., 2022; Walzer et al., 2022). In addition, the proximate causes responsible for size shifts were species‐specific: WGP effects in the predator, TGP effects in the prey. Predators may have the ability to avoid heat stress by moving to sites differing from the parental environment, whereas the prey tends to stay put, thereby experiencing similar heat stress as their parents. The decisive condition for adaptive TGP, the matching of parental and offspring environments (Burgess & Marshall, 2011), seems to be less satisfied for the actively hunting predator females, reflected by their faster movement (Walzer, personal observation) and higher metabolic rates compared to their less mobile prey (Thurling, 1980). This assumption is supported by the high escape rates of the predatory females compared to the prey. Thus, we argue that the different mechanisms modifying predator and prey body sizes could be adaptations to their respective lifestyles.

As for the majority of ectothermic species, the shift in body size is in agreement with the temperature–size rule (small size under high temperatures) (Angilletta Jr, 2009; Atkinson, 1994) for the predator but not for the prey. Evidence for reverse responses of ectotherms (large size under high temperatures) comes from insects (Fu et al., 2016; Kingsolver et al., 2007; Walters & Hassall, 2006), mites (Walzer et al., 2020), lizards (Sheridan, Martin, et al., 2022a), and frogs (Sheridan, Mendenhall, & Yambun, 2022b). We may therefore ask which phenotype (small versus large) provides benefits under extreme heat waves? First, only few studies evaluated the relationship between size and heat tolerance, with diverging results. Body size was positively correlated with heat resistance in dung flies (Esperk et al., 2016) and ants (Wendt & Verble‐Pearson, 2016), and negatively with adult dragonflies and damselflies (Bota‐Sierra et al., 2022), whereas size did not affect heat resistance in fruit flies (Condon et al., 2015; Jenkins & Hoffmann, 1994; Popa‐Báez et al., 2020) and house flies (Kjærsgaard et al., 2015). However, since climate warming will not only increase the severity of heat waves but also dry spells (Sarhadi et al., 2018), desiccation risk may constitute an additional climatic stressor for ectotherms. Large individuals are expected to benefit from such climatic conditions because of their lower surface:volume ratio, which reduces water loss rates more efficiently than in small individuals (Bujan et al., 2016; Gardner et al., 2011). Experimental evidence for this assumption comes from dung beetles (Nervo et al., 2021), fruit flies (Popa‐Báez et al., 2020), isopods (Yilmaz et al., 2021), lizards (Sheridan, Martin, et al., 2022a; Wogan & Richmond, 2015), and frogs (Sheridan, Mendenhall, & Yambun, 2022b). Accordingly, large prey females should be better adapted to dry and hot weather conditions compared with the smaller predator females. Second, a potential cost of large body size may arise when a large body size is coupled with viability costs of longer juvenile development because of decreased juvenile survival and a later start of reproduction (Blanckenhorn, 2000). However, the large body size of female prey is not the result of prolonged juvenile development under extreme heat waves but rather due to increased growth rates (Tscholl et al., 2022, Walzer et al., 2022). Third, in many species, a high reproductive output correlates with a larger female size (Blanckenhorn, 2000), which also seems to be the case for *T. urticae* in our study, although the association between body size and oviposition rate was not very strong. In contrast, we did not detect any costs or benefits of small female size in the predator because consumption, egg number, and egg size were found to be insensitive to female body size. Summing up, we argue that the large female size of the prey should provide benefits over the predator with respect to reproductive investment, resistance to predator attacks, desiccation, and/or heat resistance. This may jeopardize biological control of spider mites by means of predatory mites when climate warming proceeds (Weintraub & Palevsky, 2008).

### 4.4 Potential impacts on population dynamics under natural conditions

Based on laboratory data related to juvenile development and fecundity, the predator seems to have several disadvantages under extreme heat waves compared to prey: (i) lower gain in developmental rates; (ii) shifts in predator–prey body size ratios in favor of the prey; (iii) higher total losses of reproductive females; and (iv) less reproductive investment (Tscholl et al., 2022, 2023; Walzer et al., 2022). Nonetheless, the consequences of these heat wave‐induced trait changes cannot be directly extrapolated to the population dynamics of *P. persimilis* and *T. urticae* because several factors present under natural conditions are missing in the lab experiments:

1. The occurrence of drought periods often correlates with heat waves (Sarhadi et al., 2018), which can strongly limit the food resources for herbivorous prey and, as a consequence, also for the predator (Adamo et al., 2012). The predator, however, should suffer more from food limitations than its prey because it is a food‐specialist in contrast to its generalist phytophagous prey (Mcmurtry & Croft, 1997). Besides, food limitation is more likely to occur in natural habitats compared to agro‐ecosystems where management methods (e.g. large monocultures, irrigation systems) aim at maximizing crop growth.
2. The capacity for population increase in predators and prey with short generation times mainly depends on their developmental rates and less on their reproductive output (Sabelis, 1985c). Thus, lower reproductive investment might have minor consequences for the population growth of the predator.
3. A large proportion of the female predators (74%) escaped during lab experiments. Under natural conditions, these individuals may not just be lost but might influence population dynamics elsewhere in the ecosystem (Nachman, 2001). Interestingly, escape rates were high irrespective of heat wave type, indicating that even mild stress may elicit an escape response. The daily escape rates peaked on the third day when prey was still plentiful. Hence, it seems unlikely that lack of prey in itself was responsible for the high emigration rates. Since metabolic costs increase with higher temperatures (Brown et al., 2004), the females will try to compensate for growing energy expenditures by increasing their predation rate. To achieve this, the females can either increase their speed of movement or invest more time in searching for prey, or do both. In a natural system, such behavior will increase a female's chance of finding a more suitable patch to settle in, whereas in our experimental set‐up, leaving the detached leaves could be lethal. Though progressive heat stress can be counteracted by increasing foraging efficiency, there is an upper limit to how fast a female can move and how much time it can spend searching. Once this limit is reached, the female will build‐up an energy deficit, which eventually will force her to slow down pace, explaining why the escape rates initially increased and then started to decline after about 3 days of exposure to heat wave conditions. However, instead of searching for a physiological explanation for the observed emigration pattern, it is tempting to consider it as an example of the “Milker” strategy (Baalen & Sabelis, 1995). The so‐called “milkers” are female predators that abandon a prey patch with sufficient prey to subsidize their offspring (see also Revynthi et al., 2018).
4. Under natural conditions, predators usually stay on the underside of leaves, where shade provides protection against sun light. However, if this is not enough, they may escape by moving to the lower parts of the foliage, where thermal conditions are typically more suitable than in the upper parts (Vela et al., 2017). In contrast, the more heat‐resistant spider mites usually move from the lower to the upper canopy as part of the colonization process because here they find the youngest leaves with high nutritional value (Walzer et al., 2009). Consequently, the highest densities of spider mites are usually found on the top leaves (Hussey & Parr, 1963). Hence, heat waves may lead to vertical separation of prey and predators, which may seriously affect the predators' chances of controlling the prey (Vela et al., 2017). Theoretically, predator females may evade lethal temperatures by means of two different strategies: First, to change their diurnal movement patterns by using lower plant parts for time‐limited recovery after heat stress, and then return to high‐quality prey patches when the temperature drops in the evening hours. Alternatively, they may stay permanently in the lower plant parts where prey density is low. In the first case, they have access to abundant food, at least during part of the day, thereby limiting the growth rate of their prey. On the other hand, it may be time‐consuming and energetically costly for the small and wingless predators to perform such daily vertical movements (Croft & Jung, 2001). In the latter case, the predators will save energy but have little impact on the prey population.

## 5 CONCLUSIONS

Our lab study reveals that more intense heat waves can lead to shifts in the total losses (escapers + mortality), behavior, food consumption, quantity, and quality (i.e., size) of deposited eggs, and body sizes of predator and prey offspring, deriving from heat wave‐exposed parents. In accordance with the trophic sensitivity hypothesis (Cheng et al., 2016; Voigt et al., 2003), the predator seems to be more heat‐sensitive (high escape rates, smaller body sizes, and smaller deposited eggs) than its prey (low total losses, larger body size, and increased number of deposited eggs) under extreme heat waves. These species‐specific changes could be explained by their different lifestyles. The mobile predator seems to be selected for spatiotemporal avoidance of heat‐exposed locations, as indicated by their high escape rates. These findings also prevent the separation of genetic effects from plastic responses in the predator. In contrast, the low total losses indicate that the physiological adaptations are plastic responses of the more sessile prey under extreme heat waves. Finally, experiments at the population or ecosystem level are needed to reveal to what extent our findings have consequences for the biological control of spider mites by means of predatory mites under intensified heat waves due to climate change.

## AUTHOR CONTRIBUTIONS

**Thomas Tscholl:** Data curation (equal); investigation (equal); methodology (equal); visualization (equal); writing – original draft (equal); writing – review and editing (equal). **Gösta Nachman:** Formal analysis (equal); writing – review and editing (equal). **Bernhard Spangl:** Formal analysis (equal); writing – review and editing (equal). **Ida Scalmani:** Investigation (equal). **Andreas Walzer:** Conceptualization (equal); data curation (equal); investigation (equal); methodology (equal); supervision (equal); writing – original draft (equal); writing – review and editing (equal).

## CONFLICT OF INTEREST STATEMENT

The authors declare no competing interests.

## Supporting information


Data S1.

## Data Availability

The data are uploaded as supporting information.

## APPENDIX A

**TABLE A1:** Diurnal variations of temperature and relative humidity (RH) mimicking mild and extreme heat waves at long‐day conditions (L:D = 16 h:8 h).

Time of the day	Mild heat wave		Extreme heat wave	
	Temperature (°C)	RH (%)	Temperature (°C)	RH (%)
00:00–02:00	18.0	75.0	24.0	65.0
02:00–04:00	17.0	80.0	23.0	70.0
04:00–06:00	16.0	85.0	22.0	75.0
06:00–08:00	19.0	75.0	25.0	65.0
08:00–10:00	27.0	60.0	33.0	50.0
10:00–12:00	30.0	50.0	36.0	50.0
12:00–14:00	32.0	50.0	38.0	50.0
14:00–16:00	29.0	55.0	35.0	50.0
16:00–18:00	24.0	65.0	30.0	55.0
18:00–20:00	22.0	70.0	28.0	60.0
20:00–22:00	19.0	75.0	25.0	65.0
22:00–00:00	18.0	75.0	24.0	65.0
MEAN	22.6	67.9	28.6	60.0

**TABLE A2:** Effects of parental (F0) and offspring (F1) heat waves (mild or extreme) and their interaction on the survival and escape rates of adult females of *P. persimilis* and *T. urticae*.

Parameter	Source of variation	*P. persimilis*		*T. urticae*	
		$\chi^{2}$	*p*	$\chi^{2}$	*p*
Escaping	F0	2.582	.108	0.073	.787
	F1	1.947	.163	6.403	**.011**
	F0*F1	5.04	.169	6.867	.076
Survival	F0	1.297	.255	0.002	.967
	F1	1.357	.244	2.845	.092
	F0*F1	2.331	.507	3.131	.372

*Note*: *p*‐Values in bold are significant at the 5% level.

**TABLE A3:** Averages and 95% confidence limits (CL) of the food consumption in eggs/day (*P. persimilis*) or mm^2^ leaf area/day (*T. urticae*) of the four treatment groups.

Parental treatment (F0)	Offspring treatment (F1)	*P. persimilis*			*T. urticae*		
		Avg.	Lower CL	Upper CL	Avg.	Lower CL	Upper CL
Mild	Mild	22.90	22.23	23.57	13.10	11.92	14.28
Mild	Extreme	22.86	21.10	24.62	15.30	14.05	16.54
Extreme	Mild	22.97	21.66	24.26	9.82	8.69	10.94
Extreme	Extreme	23.94	22.60	25.28	13.79	12.67	14.92

*Note*: Only females surviving the entire experimental period (10 days) were used in the calculation.

**TABLE A4:** Effects of female age (*t*), ln(*t*), parental (F0), and offspring heat waves (F1) and the interactions F1**t* and F1*ln(*t*) on the within‐subject oviposition rates of *P. persimilis* and *T. urticae*.

Source of variation	*P. persimilis*		*T. urticae*	
	$\chi^{2}$	*p*	$\chi^{2}$	*p*
ln(*t*)	70.80	**<.0001**	70.07	**<.0001**
*t*	60.75	**<.0001**	60.57	**<.0001**
F1	29.73	<**.0001**	11.90	**.0006**
F0	1.30	.2539	1.46	.2268
F1*ln(*t*)	13.97	**.0002**	2.35	.1249
F1**t*	5.95	**.0147**	0.38	.5427

*Note*: *p*‐Values in bold are significant at the 5% level.

**TABLE A5:** Averages, sample size, and 95% confidence limits (CL) of the egg size (in ×10^6^ μm^3^) of different groups of male and female *P. persimilis* and *T. urticae*.

Sex	Treatment	*P. persimilis*				*T. urticae*			
		*N*	Avg. volume	Lower 95% CL	Upper 95% CL	*N*	Avg. volume	Lower 95% CL	Upper 95% CL
Female	EE	151	3.869	3.822	3.917	290	1.143	1.130	1.157
	ME	169	3.740	3.710	3.770	249	1.155	1.143	1.167
	EM	167	4.193	4.139	4.248	247	1.239	1.222	1.256
	MM	179	4.233	4.171	4.297	340	1.243	1.130	1.266
Male	EE	46	3.751	3.679	3.824	390	1.120	1.108	1.131
	ME	63	3.687	3.620	3.757	316	1.124	1.110	1.138
	EM	60	4.038	3.948	4.128	241	1.165	1.149	1.181
	MM	67	4.038	3.929	4.147	206	1.176	1.159	1.194

*Note*: F1 females, originating from parents reared under mild (M) or extreme (E) heat waves, and F1 females exposed to mild (M) or extreme (E) heat waves are labeled by the first and second upper case letters, respectively: MM, ME, EM, and EE.

**TABLE A6:** Effects (GEE analysis) of offspring heat waves (F1), female age (*t*), *t* ^2^, and F1**t* on the ln‐transformed volumes of female and male eggs of *P. persimilis* and *T. urticae*.

Sex of eggs	Source of variation	*P. persimilis*		*T. urticae*	
		$\chi^{2}$	*p*	$\chi^{2}$	*p*
Females	F1	11.43	**.0007**	0.83	.3628
	*t*	17.98	**<.0001**	42.28	**<.0001**
	*t* ^2^	10.65	**.0011**	17.70	**<.0001**
	*t**F1	13.83	**.0002**	19.74	**<.0001**
Males	F1	2.48	.1153	0.88	.3479
	*t*	7.97	**.0047**	47.81	**<.0001**
	*t* ^2^	5.76	**.0164**	25.12	**.0001**
	*t**F1	11.72	**.0006**	22.35	**<.0001**

*Note*: *p*‐Values in bold are significant at the 5% level.

**TABLE A7:** Effects (GEE analysis) of female age (*t*) and *t* ^2^ on the ln‐transformed volumes of female and male eggs of *P. persimilis* and *T. urticae* laid by females exposed to either mild or extreme heat waves.

F1 Treatment	Sex of eggs	Source of variation	*P. persimilis*		*T. urticae*	
			$\chi^{2}$	*p*	$\chi^{2}$	*p*
Mild	Males	*T*	12.04	**.0005**	23.17	**<.0001**
		*t* ^2^	8.57	**.0034**	10.79	**.0010**
	Females	*T*	17.47	**<.0001**	33.63	**<.0001**
		*t* ^2^	11.82	**.0006**	18.61	**<.0001**
Extreme	Males	*T*	0.10	.7563	23.62	**<.0001**
		*t* ^2^	0.15	.6960	14.96	**.0001**
	Females	*T*	0.64	.4243	8.79	**.0030**
		*t* ^2^	0.17	.6760	1.87	.1790

*Note*: *p*‐Values in bold are significant at the 5% level.
